# Improvement of locomotive syndrome with surgical treatment in patients with degenerative diseases in the lumbar spine and lower extremities: a prospective cohort study

**DOI:** 10.1186/s12891-020-03547-0

**Published:** 2020-08-03

**Authors:** Satoshi Kato, Yuki Kurokawa, Tamon Kabata, Satoru Demura, Hidenori Matsubara, Yoshitomo Kajino, Yoshiyuki Okamoto, Hiroaki Kimura, Kazuya Shinmura, Kentaro Igarashi, Takaki Shimizu, Noritaka Yonezawa, Noriaki Yokogawa, Hiroyuki Tsuchiya

**Affiliations:** 1 Department of Orthopaedic Surgery, Graduate School of Medical Sciences, Kanazawa University, 13-1 Takara-machi, Kanazawa, 920-8641 Japan; 2 Department of Orthopaedic Surgery, Yawata Medical Center, 12-7 Yawata, Komatsu, 923-8551 Japan; 3 Department of Orthopaedic Surgery, Kanazawa Red Cross Hospital, 2-251 Mima, Kanazawa, 921-8162 Japan

**Keywords:** Degenerative disease, Improvement, Locomotive syndrome, Lower extremity, Lumbar spine, Outcome, Surgery

## Abstract

**Background:**

The epidemiology, risk factors, and prevention of locomotive syndrome (LS) have been reported. However, the number of clinical studies about the efficacy of LS treatment, including surgery, has been limited. This study aimed to evaluate LS and its improvement in patients undergoing surgeries for degenerative disease of the lumbar spine and lower extremities, and to discuss the effects of surgery on LS and the issues of LS assessment in these patients.

**Methods:**

We enrolled 257 patients aged ≥60 years that underwent surgery for degenerative diseases of the lumbar spine and lower extremities and agreed to participate in the preoperative and 6- and 12-month postoperative LS examinations. According to the disease location, patients were divided into the lumbar (*n* = 81), hip (*n* = 106), knee (*n* = 43), and foot and ankle (*n* = 27) groups. Patients underwent LS risk tests, including the stand-up test, two-step test, and 25-Question Geriatric Locomotive Function Scale (GLFS-25) assessment.

**Results:**

The preoperative prevalence of LS stage 2 was 95%. Only the hip group showed significant improvements in the stand-up test. The knee group showed the worst results in the stand-up and two-step tests at all time points. All four groups had significant improvements in GLFS-25 scores. Approximately 40% of all patients had improvement in their LS stage postoperatively. However, > 90% of the patients in the knee group had LS stage 2 postoperatively.

**Conclusion:**

Nearly all elderly patients requiring surgeries for degenerative diseases of the lumbar spine and lower extremities had advanced conditions (LS stage 2). Surgeries could be beneficial in alleviating LS. The LS stage 3 criteria should be established, and the use of the GLFS-25 assessment can be appropriate for advanced LS patients with severe musculoskeletal diseases requiring surgeries.

## Background

Decreased mobility function is an inevitable outcome of aging, with an increasing proportion of elderly individuals requiring care owing to mobility impairments [1, 2]. Considering that the increasing health care costs associated with our aging societies imposes a growing economic burden, extending healthy life expectancy and reducing health disparities are urgent tasks. Thus, the Japanese Orthopaedic Association (JOA) introduced the concept of locomotive syndrome (LS) [3]. LS is defined as a decline in locomotor function due to a musculoskeletal disorder, often leading to the need for nursing care [3–5]. Multiple factors have been associated with LS, including osteoporosis, osteoarthritis, and sarcopenia [6, 7]. The JOA developed an LS risk assessment in 2013, which consisted of three tests, with a grading system introduced in 2015, which categorizes LS into stage 1 or 2 [8]. There are many LS studies examining its epidemiology, risk factors, and prevention [9–13]. Treatment, prevention, and early identification of locomotor impairment are important to improve functional outcomes with aging. However, the number of clinical studies about the efficacy of LS treatment, including surgical treatments, remains limited [14]. The points of issue of LS evaluation have not been examined in patients with severe LS requiring surgical treatment for degenerative disease in the lumbar spine and lower extremities, which are the main factors associated with LS.

This study aimed to evaluate the LS stage and its improvement in the patients undergoing surgeries for degenerative disease of the lumbar spine and lower extremities, and to discuss the efficacy of surgeries and the issues of LS evaluation in these patients.

## Methods

### Ethics statement

The ethics committees of Kanazawa university hospital and our affiliated hospitals (Yawata Medical Center and Kanazawa Red Cross Hospital) approved this prospective study (No. 2015–109). Written informed consent was obtained from each subject.

### Subjects

The clinical data of 399 patients, who underwent surgery for degenerative diseases of the lumbar spine and lower extremities at our three hospitals between January 2016 and December 2018 and who agreed to participate in the pre- and postoperative examinations, were prospectively collected. The lumbar diseases included lumbar spinal stenosis with or without spondylolisthesis that were treated with posterior decompression or short-segment spinal fusion surgeries. The hip joint diseases included hip osteoarthritis and avascular necrosis of the femoral head that were treated with total hip arthroplasty (THA). The knee joint diseases included knee osteoarthritis and spontaneous osteonecrosis of the knee that were treated with total knee arthroplasty (TKA), unicompartmental knee arthroplasty, or high tibial osteotomy. The foot and ankle diseases included joint osteoarthritis of the ankle and midfoot, and the foot deformities that were treated with arthrodesis or corrective osteotomies of the ankle and foot. According to disease locations, patients were divided into the lumbar, hip, knee, and foot and ankle groups.

Patients aged < 60 years were excluded because LS prevalence is closely associated with age [9] and the hip group included a significant portion of younger patients. Patients with incomplete data at the three time points or who underwent another surgery for spinal or extremity disorders during the study period (until 12 months postoperatively) were also excluded. In the three hospital, the rehabilitation protocol was standardized. Every patient had postoperative rehabilitation program to maintain or improve muscle strength, range of motion of the operated joint, and activity of daily living for about 2 weeks after surgery until discharge from the hospitals. After discharge, they did not have additional rehabilitation by physical therapist. In the patients who underwent lumbar, hip, and knee surgeries including high tibial osteotomy, full weight bearing was allowed immediately after surgery. In the patients who underwent ankle and midfoot surgeries, it was allowed after a 4-week postoperative period of non-weight bearing. In the patients who underwent forefoot surgeries, full weight bearing on the forefoot was allowed after a 4-week postoperative period of weight bearing only on the heel.

### Outcome measures

We evaluated the LS stage of these patients at a few days preoperatively and at 6 and 12 months postoperatively using the three LS risk tests [4] proposed by the JOA. The three tests, namely, the stand-up test, two-step test, and 25-Question Geriatric Locomotive Function Scale (GLFS-25) assessment [15] were performed according to the JOA guidelines [4]. The risk levels for LS of each test and their total assessment were classified as stage 0, 1, or 2. The stand-up test quantifies lower limb strength by evaluating an individual’s ability to stand from the sitting position, using single- or double-leg stance, from four different heights of 10, 20, 30, and 40 cm [4]. Nine performance scores are possible, as previously described [16]: 0 (inability to stand); 1, 2, 3, or 4 (stand using both legs from a height of 40, 30, 20, and 10 cm, respectively); and 5, 6, 7 and 8 (stand using one leg from a height of 40, 30, 20, and 10 cm, respectively). The scores < 3 and < 5 were classified as LS stages 2 and 1, respectively. The two-step test measures the maximum stride length, normalized to the patient’s height, over two strides [4]. This test provides a measure of lower limb strength, flexibility, and standing balance [8]. The scores of < 1.1 and < 1.3 were classified as LS stages 2 and 1, respectively. The GLFS-25 is a self-reported assessment of locomotor function over the past month [15], with each item scored on a 5-point Likert scale, ranging from ‘0’ (no impairment) to ‘4’ (severe impairment). The total score can range from ‘0’ to ‘100’, with an increasing score indicative of greater severity of locomotor impairment. A GLFS-25 score of ≥16 and ≥ 7 were classified as LS stages 2 and 1, respectively.

The worst LS stage obtained on each of these three tests was used to classify a patient’s total LS stage for analysis [4]. In this study, LS improvement was evaluated based on the results of the three tests. LS improvement was defined as the postoperative downgrading of LS stage in each test compared with the preoperative LS stage. When the preoperative LS stage was zero (non-LS) in rare cases, the improvement was defined as the postoperative improved measurements of the tests.

### Statistical analysis

Continuous variables are expressed as mean ± standard deviation, and ordinal variables are expressed as median (interquartile range). A repeated measures analysis of variance, followed by a Tukey post hoc test, was used to evaluate within-group differences in the variables of the three locomotive risk tests. The Tukey-Kramer honestly significant difference test was used to compare the variables among the four groups. For countable.

data were expressed as a percentage, comparisons between groups were performed using the chi-square test. All statistical analyses were performed using SPSS version 25 (IBM Corp., Armonk, NY, USA). *P*-value < 0.05 was considered statistically significant.

## Results

Finally, 257 patients were included and evaluated in the study [lumbar group, *n* = 81; hip group, *n* = 106; knee group, *n* = 43; and foot and ankle group, *n* = 27]. The knee group was significantly older than the hip and foot and ankle groups. The lumbar group had a lower female ratio than the hip group. The hip group had a lighter weight and a lower body mass index than the knee and foot and ankle groups (Table 1). The detailed information of disease pathologies and surgeries of the four groups were presented in Table 1. In THA, anterior and posterior approaches were used in 62 and 44 patients, respectively. In knee surgeries, anterior approach was used in all patients.

**Table 1:** Background characteristics of the four groups

Groups	Lumbar (***n*** = 81)	Hip (n = 106)	Knee (n = 43)	Foot & Ankle (***n*** = 27)
**Demographic data**				
Age (year), mean (SD)	71.5 (6.9)	69.2 (6.3)^**§**^	73.7 (7.1) ^**#,¶**^	68.9 (6.0)^**§**^
Sex, female, n (%)	48 (59.3%)^**#**^	93 (87.7%)^**†**^	35 (81.4%)	21 (77.8%)
Height (cm), mean (SD)	157.9 (8.8)	154.1 (8.1)	154.7 (8.3)	156.2 (9.9)
Weight (kg), mean (SD)	56.3 (11.8)	55.1 (10.0)^**§,¶**^	60.4 (10.9) ^**#**^	62.6 (12.3) ^**#**^
BMI (kg/m^2^), mean (SD)	23.9 (3.4)	23.2 (3.7)^**§,¶**^	25.1 (3.7) ^**#**^	25.5 (3.7) ^**#**^
Disease pathology (n)	LSS (81)	Hip OA (100) ANFH (6)	Knee OA (42) SONK (1)	Ankle OA (13) Midfoot OA (4) Foot deformity (10)
Surgery (n)	Decompression (55) Short-segment spinal fusion (26)	THA (106)	TKA (33) HTO (8) UKA (2)	Arthrodesis (15) Corrective osteotomy (12)
**Preoperative LS status**				
Prevalence of LS (stage 1 and 2), %	100%	100%	100%	100%
Prevalence of LS stage 2, %	95.1%	95.3%	100%	85.2%
**Inclusion Criteria**				
・Patients underwent surgeries for degenerative diseases of the lumbar spine or lower extremities in our hospitals from January 2016 to December 2018.				
・Patients agreed to participate in the pre- and postoperative physical examinations.				
**Exclusion Criteria**				
・Patients were younger than 60 years of age.				
・Patients had incomplete data at the three points (preoperatively and at 6 and 12 months postoperatively).				
・Patients underwent another surgery for spinal or extremity disorders during the study period.				

^**†**^P < 0.05 versus Lumbar group, ^**#**^*P* < 0.05 versus Hip group, ^**§**^P < 0.05 versus Knee group, ^**¶**^P < 0.05 versus Foot & Ankle group,

*ANFH* avascular necrosis of the femoral head; *BMI* body mass index; *HTO* high tibial osteotomy; *LS* locomotive syndrome; *LSS* lumbar spinal stenosis; *OA* osteoarthritis; *SD* standard deviation; *SONK* spontaneous osteonecrosis of the knee; *THA* total hip arthroplasty; *TKA* total knee arthroplasty; *UKA* unicompartmental knee arthroplasty

**Fig. 1:**
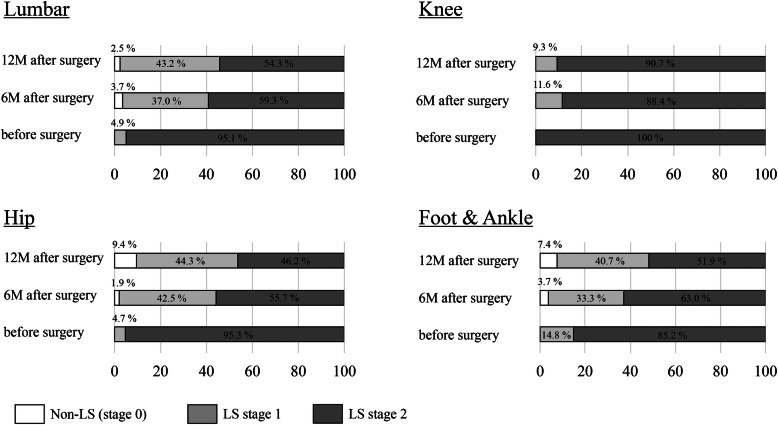
The distribution of locomotive syndrome stage based on the total assessment preoperatively and at 6 and 12 months postoperatively among the four surgical groups

Table 2 showed the prevalence of LS stage 2 and LS (a total of stages 1 and 2) based on each of the three LS risk tests and total assessment among the four groups preoperatively and at 6 and 12 months postoperatively. Preoperatively, 244 (94.9%) of the 257 patients were classified as having total LS stage 2, and all patients were classified as having LS. All 43 patients in the knee group were classified as having stage 2 preoperatively state. Large discrepancies in the pre- and postoperative prevalence of LS stage 2 among the four groups were observed in the stand-up and two-step test results, with the worst results being observed in the knee group (Table 2). Contrarily, the discrepancies among the four groups were decreased in the results of the GLFS-25. The prevalence of LS stage 2 at 6 and 12 months postoperatively was 63.0% (162 patients) and 56.8% (146 patients), respectively. The proportion of patients with postoperative LS stage improvement at 6 and 12 months postoperatively was 35.0% (90 patients) and 39.7% (102 patients), respectively. At 12 months postoperatively, approximately half of the lumbar, hip, and foot and ankle groups were classified as having total LS stage 0 or 1 (not stage 2). However, > 90% of the knee group were classified as having LS stage 2 (Table 2 and Fig. 1).

**Table 2:** Prevalence of LS stage 2 and LS based on each of the three LS risk tests and total assessment

Groups	Total (***n*** = 257)	Lumbar (***n*** = 81)	Hip (***n*** = 106)	Knee (***n*** = 43)	Foot & Ankle (***n*** = 27)
**Total assessment: prevalence of LS stage 2, % (that of LS [LS stage 1 and 2], %)**					
Before surgery	94.9 (100)	95.1 (100)	95.3 (100)	100 (100)	85.2 (100)
6 months after surgery	63.0 (97.7)	59.3 (96.3)	55.7 (98.1)	88.4 (100)	63.0 (96.3)
12 months after surgery	56.8 (94.6)	54.3 (97.5)	46.2 (90.6)	90.7 (100)	51.9 (92.6)
**Stand-up test: prevalence of LS stage 2, % (that of LS [LS stage 1 and 2], %)**					
Before surgery	47.5 (84.4)	21.0 (79.0)	60.4 (87.7)	83.7 (93.0)	18.5 (74.1)
6 months after surgery	36.2 (86.4)	22.2 (86.4)	33.0 (86.8)	86.0 (95.3)	11.1 (70.4)
12 months after surgery	33.5 (80.9)	17.3 (79.0)	30.2 (77.4)	86.0 (97.7)	11.1 (74.1)
**Two-step test: prevalence of LS stage 2, % (that of LS [LS stage 1 and 2], %)**					
Before surgery	58.8 (89.9)	44.4 (79.0)	71.7 (96.2)	76.7 (95.3)	22.2 (74.1)
6 months after surgery	39.3 (76.7)	37.0 (67.9)	37.7 (79.2)	53.5 (83.7)	29.6 (81.5)
12 months after surgery	31.1 (69.3)	28.4 (61.7)	29.2 (69.8)	53.5 (86.0)	11.1 (63.0)
**GLFS-25: prevalence of LS stage 2, % (that of LS [LS stage 1 and 2], %)**					
Before surgery	90.3 (99.6)	92.6 (100)	91.5 (100)	93.0 (100)	74.1 (96.3)
6 months after surgery	43.6 (77.8)	46.9 (77.8)	33.0 (69.8)	60.5 (93.0)	48.1 (85.2)
12 months after surgery	39.7 (75.9)	45.7 (84.0)	28.3 (62.3)	53.5 (90.7)	44.4 (81.5)

*GLFD-25* 25-Question Geriatric Locomotive Function Scale; *LS* locomotive syndrome

**Fig. 2:**
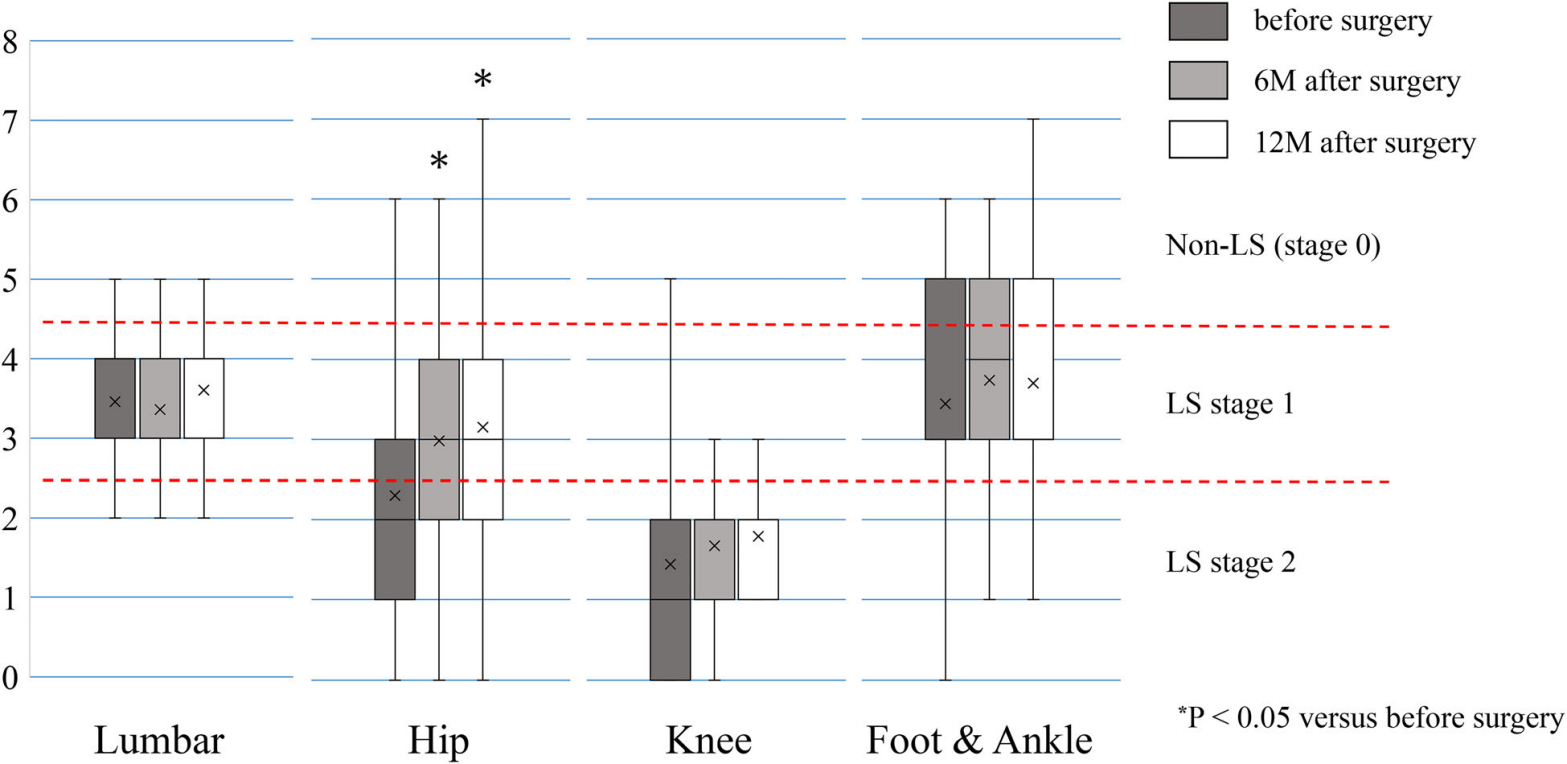
The distribution of the stand-up test results preoperatively and at 6 and 12 months postoperatively among the four surgical groups

Table 3 shows the data distribution the stand-up test among the four groups at all time points. The knee group had the worst results at all time points, with measurement values being lower than were those of the other three groups. Only the hip group had significant improvements in the measurement values at 6 and 12 months postoperatively compared with the preoperative values (*p* < 0.05; Fig. 2). The proportion of the hip group with postoperative LS improvement in the stand-up test at 6 and 12 months postoperatively was 40.6% (43 patients) and 44.3% (47 patients), respectively (Table 3); whereas, that of the knee group was the lowest with only 9.3% (4 patients) of patients with improvements at both 6 and 12 months postoperatively.

**Table 3:** Data and LS improvement of the stand-up test in the four groups

Groups	Lumbar (n = 81)	Hip (n = 106)	Knee (n = 43)	Foot & Ankle (n = 27)
**Score of the stand-up test**				
**Before surgery, mean (median)**	3.46 (3.58) ^**#,§**^	2.30 (2.11) ^*,§,¶^	1.44 (1.17) ^*, #,¶^	3.44 (3.47) ^**#,§**^
**6 months after surgery, mean (median)**	3.36 (3.32)^**§**^	2.98 (2.97)^**§,¶**^	1.67 (1.56) ^*, #,¶^	3.74 (3.69) ^**#,§**^
**12 months after surgery, mean (median)**	3.60 (3.54)^**§**^	3.15 (3.20)^**§**^	1.79 (1.68) ^*, #,¶^	3.70 (3.59)^**§**^
**Prevalence of patients with LS improvement based on the stand-up test**				
**6 months after surgery, %**	11.1%	40.6%	9.3%	22.2%
**12 months after surgery, %**	22.2%	44.3%	9.3%	18.5%

^*^P < 0.05 versus Lumbar group, ^**#**^*P* < 0.05 versus Hip group, ^**§**^P < 0.05 versus Knee group, ^**¶**^*P* < 0.05 versus Foot & Ankle group,

*LS* locomotive syndrome

The scores < 3 and < 5 were classified as LS stages 2 and 1, respectively

LS improvement was defined as the postoperative downgrade of LS grade in each of the tests

When the preoperative LS grade was zero in the rare cases, the improvement was defined as the postoperative improved measurements of the tests

**Fig. 3:**
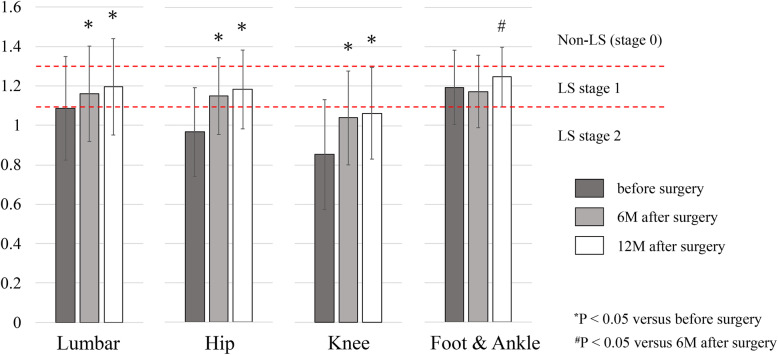
The distribution of the two-step test results preoperatively and at 6 and 12 months postoperatively among the four surgical groups

Table 4 shows the data distribution of the two-step test among the four groups at the three time points. The knee group had the worst results in the two-step test at all time points, with measurement values being lower than were those of the lumbar and foot and ankle groups preoperatively and lower than were those of the other three groups at 6 and 12 months postoperatively. The lumbar, hip, and knee groups had significant improvements in the measurement values of the two-step test at 6 and 12 months postoperatively compared with the preoperative values (*p* < 0.05: Fig. 3). However, the proportion of the knee group with postoperative LS improvement in the two-step test at 12 months postoperatively was limited and lower than those of the other three groups, including the foot and ankle group, because the preoperative values of the knee group was the lowest among the four groups (Table 4).

**Table 4:** Data and LS improvement of the two-step test in the four groups

Groups	Lumbar (***n*** = 81)	Hip (***n*** = 106)	Knee (***n*** = 43)	Foot & Ankle (***n*** = 27)
**Score of the two-step test**				
**Before surgery, mean (SD)**	1.09 (0.26) ^**#,§**^	0.97 (0.23) ^*,¶^	0.85 (0.28) ^*,¶^	1.19 (0.19) ^**#,§**^
**6 months after surgery, mean (SD)**	1.16 (0.24)^**§**^	1.15 (0.20)^**§**^	1.04 (0.24) ^*, #,¶^	1.17 (0.18)^**§**^
**12 months after surgery, mean (SD)**	1.20 (0.24)^**§**^	1.18 (0.20)^**§**^	1.06 (0.23) ^*, #,¶^	1.25 (0.15)^**§**^
**Prevalence of patients with LS improvement based on two-step test**				
**6 months after surgery, %**	38.3%	48.1%	27.9%	18.5%
**12 months after surgery, %**	46.9%	59.4%	34.9%	44.4%

^*^P < 0.05 versus Lumbar group, ^**#**^P < 0.05 versus Hip group, ^**§**^P < 0.05 versus Knee group, ^**¶**^P < 0.05 versus Foot & Ankle group,

*LS* locomotive syndrome; *SD* standard deviation

The scores of < 1.1 and < 1.3 were classified as LS stages 2 and 1, respectively

LS improvement was defined as the postoperative downgrade of LS grade in each of the tests

When the preoperative LS grade was zero in the rare cases, the improvement was defined as the postoperative improved measurements of the tests

**Fig. 4:**
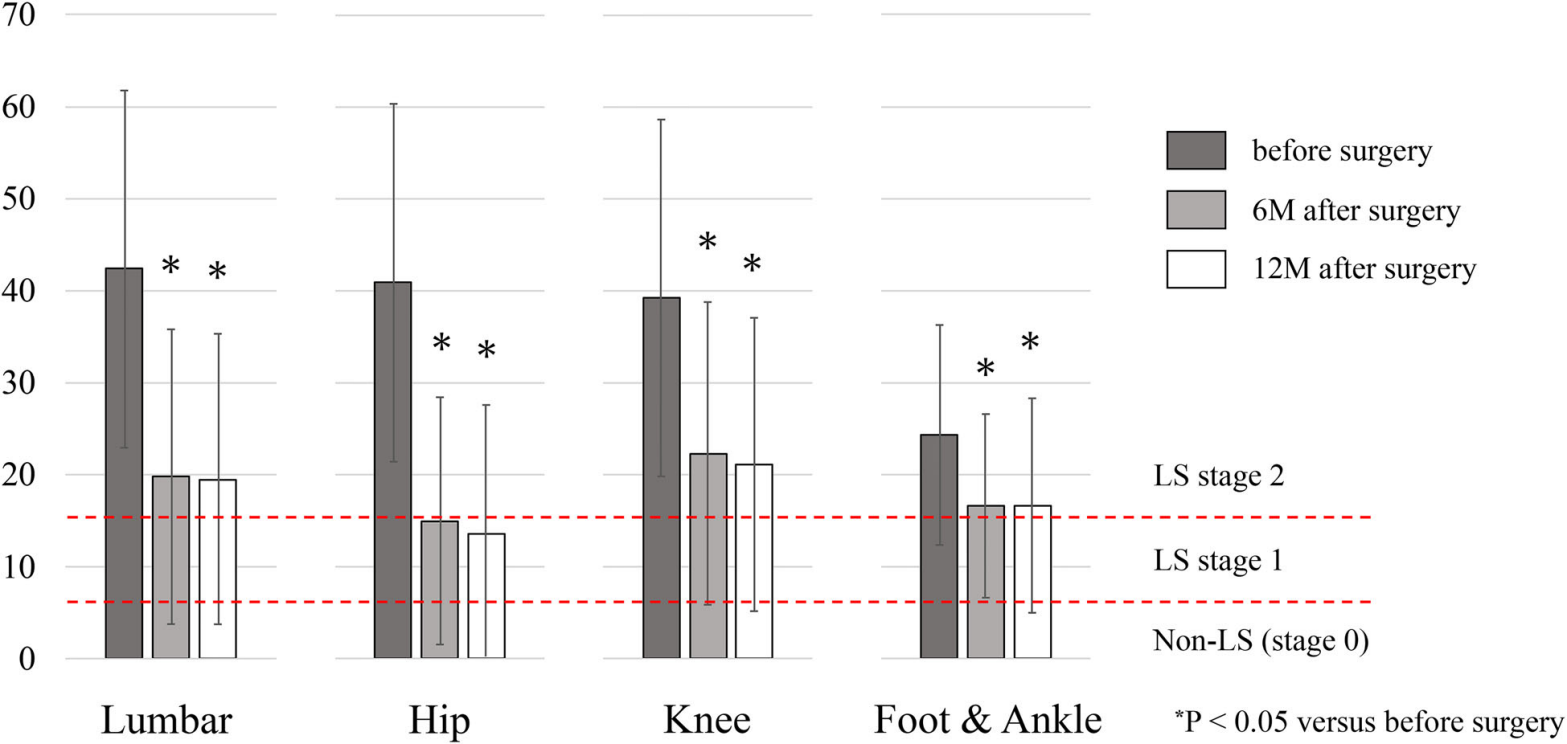
The distribution of the GLFS-25 results preoperatively and at 6 and 12 months postoperatively among the four surgical groups

Tables 5 shows the data distribution of the GLFS-25 scores among the four groups at the three time points. The lumbar group had the worst results preoperatively, whereas the knee group had the worst results at 12 months postoperatively. All four groups had significant improvements in GLFS-25 score at 6 and 12 months postoperatively compared with the preoperative values (*p* < 0.05: Fig. 4). The favorable LS improvement in GLFS-25 from 40.7 to 67.0% was observed postoperatively in all four groups. The hip group had the best results in terms of the proportion of postoperative improvement in GLFS-25 score with 61.3 and 67.0% at 6 and 12 months postoperatively, respectively (Table 5).

**Table 5:** Data and LS improvement of the GLFS-25 in the four groups

Groups	Lumbar (***n*** = 81)	Hip (n = 106)	Knee (***n*** = 43)	Foot & Ankle (n = 27)
**GLFS-25 score**				
**Before surgery, mean (SD)**	42.3 (19.4)^**¶**^	40.9 (19.5)^**¶**^	39.3 (19.4)^**¶**^	24.3 (11.9) ^*,#,§^
**6 months after surgery, mean (SD)**	19.6 (15.6)	15.0 (13.5)^**§**^	22.3 (16.5) ^**#**^	16.6 (10.0)
**12 months after surgery, mean (SD)**	19.5 (15.8) ^**#**^	13.6 (14.0)^*,§^	21.1 (16.0) ^**#**^	16.6 (11.7)
**Prevalence of patients with LS improvement based on the GLFS score**				
**6 months after surgery, %**	50.6%	61.3%	34.9%	40.7%
**12 months after surgery, %**	50.6%	67.0%	46.5%	37.0%

^*^P < 0.05 versus Lumbar group, ^**#**^P < 0.05 versus Hip group, ^**§**^P < 0.05 versus Knee group, ^**¶**^P < 0.05 versus Foot & Ankle group,

*GLFD-25* 25-Question Geriatric Locomotive Function Scale; *LS* locomotive syndrome; *SD* standard deviation

A GLFS-25 score of ≥16 and ≥ 7 were classified as LS stages 2 and 1, respectively

LS improvement was defined as the postoperative downgrade of LS grade in each of the tests

When the preoperative LS grade was zero in the rare cases, the improvement was defined as the postoperative improved measurements of the tests

## Discussion

This study evaluated the longitudinal changes in the actual data of the LS risk tests and total LS stage of the patients aged ≥60 years who underwent surgeries for the degenerative diseases in the lumbar spine and lower extremities. The results were compared among the groups divided according to the disease location. Studies evaluating the efficacy of surgeries based on the LS risk tests and LS stage are limited [14, 17]. However, this is the first study to compare these results among the disease types and to discuss the issues in LS evaluation of the patients who underwent surgeries. The Japanese large-scale population-based cohort study reported that the prevalence of LS (stages 1 and 2) and LS stage 2 were estimated at 69.8 and 25.1% (mean age, 63.9 years), respectively, in Japan [10]. In this study, the prevalence of LS and LS stage 2 in the patients aged ≥60 years (mean age, 70.7 years) who underwent surgeries for the degenerative diseases of the lumbar spine and lower extremities was 100 and 94.9%, respectively. Especially, all patients in the knee group had total LS stage 2. These diseases are common in the elderly and closely associated with LS [9, 11, 18]. This study showed that almost all elderly patients requiring surgeries for these diseases had an advanced condition of LS stage 2. Contrarily, the prevalence of LS stage 2 at 6 and 12 months postoperatively was approximately 60%. The proportion of patients with 12-month postoperative LS stage improvement was approximately 40%. Although the study showed the efficacy of surgeries for these diseases on LS improvement, the discrepancies of LS improvement were observed among the groups (Table 2 and Fig. 1).

The results of the three LS risk tests among the four groups had the following important features (Tables 3, 4 and 5, Figs. 2, 3 and 4): 1) surgery cannot improve the stand-up test results very well (improvement was observed only in the hip group), 2) surgery can improve the GLFS-25 scores very well without a remarkable discrepancy among the disease types and surgeries, 3) the knee group showed the worst results in the stand-up and two-step tests at all time points. The features were influenced by the characteristics of the diseases and surgeries. Moreover, they indicated the limitation of surgical effect and controversial point of LS assessment in patients with severe LS-related diseases requiring surgery.

In the stand-up test, only the hip group had significant improvements at 6 and 12 months postoperatively compared with the preoperative values (Fig. 2). The stand-up movement requires adequate range of motion at the joint, flexibility and balance, in addition to lower extremity muscle strength [4]. Hip and knee range of motion and knee extensor strength are especially important. Decreased pain and improved range of motion in the hip joint after THA contributed to the result. Fujita et al. reported examined the efficacy of lumbar surgery on LS and reported that the stand-up test showed a comparable distribution of stages pre- and postoperatively [14]. No other studies have described the changes in the stand-up test results by surgeries for the degenerative diseases of the lumbar spine and lower extremities. Although the lumbar, knee, and foot and ankle groups did not have improvement in the stand-up test by surgeries, the actual data distribution was different among the groups (Table 3). The median and average values of the postoperative stand-up test in the lumbar, hip, and foot ankle group were within the range of LS stage 1. However, those in the knee group were within the range of LS stage 2 (Fig. 2). The 12-month postoperative prevalence of LS stage 2 were 17.3, 30.2, 86.0, and 11.1% in the lumbar, hip, knee, and foot and ankle groups, respectively (Table 2).

In the two-step test, the lumbar, hip, and knee groups had significant improvements at 6 and 12 months postoperatively compared with the preoperative values (Fig. 3). Although the foot and ankle group did not show improvement in the two-step test by surgeries, the average values were within the range of LS stage 1 pre- and postoperatively. The results suggested that pain and limited range of motion in the ankle or foot did not influence length of stride compared with these pathologies in the hip and knee. The previous study reported that lumbar spinal stenosis decreases length of stride [19]. Contrarily, although the knee group had improvement in the two-step test by surgeries, the postoperative average value was still within the range of LS stage 2 (Fig. 3). The 12-month postoperative prevalence of LS grade 2 were 28.4, 29.2, 53.5, and 11.1% in the lumbar, hip, knee, and foot and ankle groups, respectively (Table 2).

The knee group showed the worst results of the stand-up and two-step tests pre- and postoperatively. Knee osteoarthritis is closely associated with a limited range of knee joint motion and muscle weakness of quadriceps femoris. Knee arthroplasty is generally an invasive procedure of the quadriceps femoris. The limited range of knee joint motion is still presented postoperatively. Previous studies have reported that gait speed and step length were better in THA patients than in TKA patients [20, 21]. These factors combined with preoperative knee pain significantly influenced the worst results in the stand-up and two-step tests in the knee group. The postoperative average values of the two tests were within the range of LS stage 2 only in the knee group (Figs. 2 and 3). The prevalence of LS stage 2 in the stand-up test of the knee group was 86.0%, which was the highest among the groups (Table 2). This feature resulted in the highest prevalence of LS stage 2 of the knee group (90.7%) in the total assessment (Table 2).

Contrarily, the GLFS-25 score significantly improved at 6 and 12 months postoperatively among all four groups (Fig. 4). Pre- and postoperative distributions of actual data were relatively comparable among the groups (Table 5). Thus, the 12-month postoperative prevalence of LS grade 2 were 45.7, 28.3, 53.5, and 44.4% in the lumbar, hip, knee, and foot and ankle groups, respectively (Table 2). Surgeries for the degenerative lumbar and lower extremity diseases result in pain reduction and improved function. However, in the assessment of physical function including LS risk tests, the results are significantly influenced by the disease locations and surgical type, especially in the stand-up test. The term “sarcopenia” coined by Rosenberg [22] in 1989 to draw attention to the age-related loss of muscle mass showed a similar condition. Sarcopenia was diagnosed based on the widely utilized criteria, consisting of muscle strength (grip strength), walking speed, and muscle mass measurements [23]. However, in patients with advanced diseases of the spine or lower extremities, walking speed can be significantly decreased owing to severe pain and/or muscle weakness associated with the diseases. The decreased walking speed is not directly associated with sarcopenia. Sakai et al. advocated that sarcopenia should be diagnosed only with muscle mass measurements in such patients with severe musculoskeletal diseases [24]. Similarly, we considered that the LS stage and its improvement should be determined only with the GLFS-25 scores in patients with severe musculoskeletal diseases requiring surgeries.

LS assessment was developed mainly to screen patients with age-related locomotive organ impairment and to encourage them improve their locomotive organ function with exercises [3, 4]. However, a significant number of elderly patients undergo surgeries for degenerative musculoskeletal diseases. It is also important to evaluate LS improvement with surgical treatment and its limitation. This study showed that almost all elderly patients requiring surgeries for the degenerative diseases had an advanced condition of LS stage 2. Among the patients with LS stage 2, data distribution of the LS risk tests was not comparable. A set of criteria for the most advanced LS stage 3 should be developed to effectively and correctly evaluate advanced LS patients and LS improvement with surgical treatment. To conform the ranges of LS stage 1 and 2 in the results of LS risk tests, LS stage 3 was defined based on the following criteria: a score < 1 (i.e., 0 point) in the stand-up test, which measures difficulty in standing from a 40-cm-high seat using both legs; a score < 0.9 in the two-step test; a ≥ 25 GLFS-25 score. The preoperative prevalence of LS stage 3 based on the standing-test, two-step test, GLFS-25, and total assessment would be 12.1, 30.0, 75.1, and 78.6%, respectively. Contrarily, the postoperative prevalence of LS stage 3 based on these four assessments would be decreased to 1.6, 11.3, 23.3, and 26.8%, respectively. Preoperative distribution of LS stages 0, 1, 2, and 3 based on the total assessment was 0, 5.1, 16.3, and 78.6%, respectively. Contrarily, the postoperative distribution of LS stage 0, 1, 2, and 3 was changed to 5.4, 37.7, 30.0, and 26.8%, respectively. The proportion of the patients with 12-month postoperative LS stage improvement increased to 63.0 and 67.7% based on the total assessment and GLFS-25, respectively.

The major limitation of the present study was its heterogeneous cohort, which included patients undergoing various types of surgeries for several musculoskeletal disease types. Therefore, we did not analyze the associations between the LS risk test results and the disease-specific assessments such as the JOA score, or identify factors associated with LS improvement by surgeries. Further studies are required to examine these important data in each disease condition. The knee group was significantly older than the hip and foot and ankle groups. The knee group also had a larger body mass index than the hip group. These factors could have a negative influence on the pre- and postoperative results of the knee group in the three LS risk tests to some extent. We proposed the establishment of LS stage 3 in this study. However, the detailed examination and validation will be required for it. Future studies are required to evaluate the demand in other study cohorts, and to officially establish LS stage 3 and validate the criteria. Despite this limitation, we were able to compare the LS-related results among the disease types, which was the main endpoint and one of the strong points of this study. The comparison revealed the issues of LS assessment in the advanced LS patients in this study.

## Conclusion

The preoperative prevalence of LS stage 2 in the elderly patients who underwent surgeries for the degenerative diseases of the lumbar spine and lower extremity was 95%. LS stage improvement at 12 months postoperatively was observed in 40% of the patients. We recommend that a set of criteria for LS stage 3 should be developed for the accurate assessment of the advanced LS patients, and that LS stage and its improvement should be determined using the GLFS-25 results in patients with severe musculoskeletal diseases requiring surgeries.

## Data Availability

The datasets during and/or analyzed during the current study are available from the corresponding author on reasonable request.

## Abbreviations

LS: Locomotive syndrome
GLFS-25: 25-Question Geriatric Locomotive Function Scale
JOA: Japanese Orthopaedic Association
THA: Total hip arthroplasty
TKA: Total knee arthroplasty
